# Future Perspectives on the Relevance of Auditory Markers in Prodromal Parkinson's Disease

**DOI:** 10.3389/fneur.2020.00689

**Published:** 2020-07-16

**Authors:** Evelien De Groote, Kim De Keyser, Patrick Santens, Durk Talsma, Annelies Bockstael, Dick Botteldooren, Miet De Letter

**Affiliations:** ^1^Department of Rehabilitation Sciences, Ghent University, Ghent, Belgium; ^2^Department of Neurology, Ghent University Hospital, Ghent, Belgium; ^3^Department of Experimental Psychology, Ghent University, Ghent, Belgium; ^4^Department of Information Technology, INTEC, Acoustics Research Group, Ghent University, Ghent, Belgium

**Keywords:** Parkinson's disease, auditory processing, prodromal markers, audiometry, otoacoustic emissions, dichotic listening, auditory reflexes, auditory evoked potentials

## Abstract

Research on auditory processing in Parkinson's disease (PD) has recently made substantial progress. At present, evidence has been found for altered auditory processing in the clinical stage of PD. The auditory alterations in PD have been demonstrated with low-cost and non-invasive assessments that are already used in routine clinical practice. Since auditory alterations have been reported early in disease progression, it would be highly relevant to investigate whether auditory markers could be provided in the prodromal stage of PD. In addition, auditory alterations in early stage PD might be modulated by dopaminergic medication. Therefore, the aim of this review is (1) to summarize the literature on auditory processing in PD with a specific focus on the early disease stages, (2) to give future perspectives on which audiological and electrophysiological measurements could be useful in the prodromal stage of PD and (3) to assess the effect of dopaminergic medication on potential auditory markers in the prodromal stage of PD.

## Introduction

Biomarker research in Parkinson's disease (PD) covers the development and validation of diverse clinical, biochemical, neuroimaging and genetic markers of pathological alterations, preferably in the early preclinical and prodromal stages of PD when a clinical diagnosis is not yet possible (1, 2). Valid and useful biomarkers potentially target a window of therapeutic opportunity in the early stages of the pathological process before clinical signs and symptoms emerge. Currently, evidence has been found for 16 prodromal markers in PD (3). Among these, non-motor symptoms are specifically interesting since they often precede the characteristic motor deficits in PD (4, 5). The non-motor symptoms in PD include, among others, autonomic dysfunctions, cognitive deficits, depression, sleep disorders, and sensory-perceptual alterations (6–8).

Biomarkers are specifically beneficial when they are non-invasive, easy and relatively inexpensive to administer, sufficiently available, and reliable (9, 10). These features seem explicitly present in assessments related to auditory processing, ranging from audiological toward electrophysiological measurements. At present, evidence has been found for altered auditory processing in the clinical stage of PD. The alterations range from disturbances in the processing of basic acoustic features toward the perception of affective and linguistic prosody (11, 12) and can be demonstrated with low-cost and non-invasive assessments that are already used in routine clinical practice. Since auditory alterations have also been reported in the early stages of PD, it would be highly relevant to investigate whether auditory markers can be found in the prodromal stage of PD. However, to date, no studies have assessed the potential of auditory markers in the prodromal stage of PD. In addition, auditory alterations might be modulated by dopaminergic medication. As such, assessing the effect of dopaminergic medication on auditory processing in the prodromal and early stages of PD will be important to identify pharmacological strategies to optimize auditory processing in PD. Therefore, the aim of this review is (1) to summarize the literature on auditory processing in PD with a specific focus on the early disease stages, (2) to give future perspectives on which audiological and electrophysiological measurements could be useful in the prodromal stage of PD and (3) to assess the effect of dopaminergic medication on potential auditory markers in the prodromal stage of PD.

## Methods

A stepwise approach was followed to summarize the literature on auditory processing in PD and to achieve the objectives of the current review. First, a comprehensive literature search was performed in four databases: MEDLINE (PubMed interface), Embase (Embase.com interface), Web of Science and The Cochrane Central Register of Controlled Trials (CENTRAL Cochrane Library). The search strategy was based on two separate search strategies for a systematic review covering central auditory processing in parkinsonian disorders and a systematic review covering the auditory P3 in PD. Finally, to be able to provide a comprehensive review on all auditory processing stages, elements related to peripheral auditory processing were added that were not covered by any of the two existing search strategies. The search strategy included key terms related to PD on the one hand (e.g., “Parkinson Disease,” parkinson^*^), and key terms related to auditory processing and its audiological and electrophysiological measurements on the other hand (e.g., “Hearing Threshold,” “Auditory Perception,” “Evoked Potentials Auditory, Brain Stem”). Only peer-reviewed articles in English were retained.

Second, titles and abstracts were screened to identify relevant studies. Subsequently, they were listed according to the type of audiological or electrophysiological measurement. Of these studies, Hoehn and Yahr (H&Y) staging (mean, SD, and range) and disease duration (mean, SD, and range) of the patients with PD who participated in the study, were determined. Articles were considered as “early stage PD” when the patient characteristics (of subgroups) met the following criteria: (1) all individual participants were at H&Y stage < III, (2) mean group H&Y stage was ≤ II, and (3) mean group disease duration was ≤ 6.0 years (13–15). The literature on auditory processing in all clinical disease stages of PD was synthesized for each type of audiological or electrophysiological measurement. Detailed outcomes and results of the systematic review are published elsewhere (16). Finally, a specific focus was given on the results of “early stage PD” studies.

## Audiological Measurements

### Peripheral Auditory Tests

#### Pure-Tone Audiometry

Pure-tone audiometry has been considered a gold standard in the audiological examination to evaluate hearing sensitivity. Based on the participant's response to pure-tone stimuli, hearing thresholds are measured at a frequency range of 0.250–8 kHz. The clinical aim of pure-tone audiometry is to determine the type, degree and configuration of the patient's hearing loss. In study groups with older participants, presbycusis or progressive bilateral hearing loss related to aging can be expected. Indeed, a high-frequency age-related hearing impairment has been shown in studies that used pure-tone audiometry to evaluate the hearing sensitivity of patients with PD and age-matched control participants (HCs). On the one hand, multiple studies have demonstrated the same sloping audiometric pattern in patients with PD compared to HCs, consistent with the presence of an age-related hearing loss (17–20). These studies did not find significant differences between patients with PD and HCs regarding pure-tone hearing thresholds. On the other hand, higher pure-tone hearing thresholds in patients with PD have been reported by other research groups in the middle to high frequency range (1.5–8 kHz) (21–28). The differences between study results may be influenced by the approach chosen to analyze the data (e.g., categorical vs. continuous data-analysis, consideration of confounding variables, correction for multiple comparisons). So, although a sensorineural hearing loss has been shown in patients with PD, it remains unclear whether hearing thresholds as measured with pure-tone audiometry are indeed higher in patients with PD compared to age-matched HCs.

Regarding early stage PD, Pisani et al. (23) carried out pure-tone audiometry to investigate the effect of dopaminergic treatment on hearing sensitivity in 11 previously untreated *de-novo* patients with PD. In the drug-naïve condition, a significantly higher hearing threshold was found at 2 kHz in the PD group compared to age-matched HCs. Following the initiation of dopaminergic treatment for a period between 1 and 3 months, pure-tone hearing thresholds remained unchanged in *de-novo* patients with PD. In the study of Yylmaz et al. (28), higher hearing thresholds at 4 and 8 kHz were demonstrated in patients with PD at H&Y stage II. However, in their study, patients with PD were on average 6.1 years older compared to the HC group. In a recent study, Scarpa et al. (29) also reported higher hearing thresholds at 4 to 8 kHz in patients with PD with an average disease duration of 4.8 year compared to HCs. However, the authors suggested that increased hearing thresholds in the high frequency range may not be a distinct feature of PD, as patients with multiple system atrophy demonstrated a similar sloping audiometric pattern in the same study.

#### Speech Audiometry

In addition to pure-tone audiometry, speech audiometry is also routinely administered in audiological practice. Speech audiometry compromises many tests, varying according to the type of speech material, task demands, response format and the presence of background noise. Speech recognition scores are calculated as the percentage of correct responses for each stimulus presentation level (relative to the noise). The resulting performance-intensity function is characterized in terms of its configuration and the level at which speech can be correctly identified half of the time [Speech Recognition Threshold, SRT (30)]. The clinical aim of speech audiometry is differentiating cochlear from retrocochlear lesions. Regarding speech audiometry in silence, two studies have found normal speech recognition scores in patients with PD compared to age-matched HCs (17, 31). In contrast, Vitale et al. (26) found a significantly higher SRT in patients with PD compared to age-matched HCs. In their study, significantly more patients with PD demonstrated a performance-intensity function that was suggestive of a cochlear dysfunction. The differences between study results are likely due to differences regarding hearing sensitivity of patients with PD and HCs, as measured with pure-tone audiometry. More specifically, if pure-tone hearing thresholds are increased compared to HCs, as was the case for patients with PD in Vitale et al. (26), abnormal speech recognition scores can be expected. Regarding speech audiometry in noise, no significant differences regarding speech recognition scores were reported between medicated patients with PD and HCs (17, 22). Interestingly, a modulatory effect of dopaminergic medication state was found on both speech audiometry in silence and in noise (17). The same patients with PD performed slightly better when they were tested without medication compared to when they were tested with medication.

No study has specifically focused on speech audiometry in early stage PD. As speech audiometry generally demonstrates normal results in patients with PD at more advanced disease stages, no differences are expected regarding speech perception in early stage patients with PD, provided that pure-tone hearing thresholds are within the normal range accounting for age and gender.

#### Otoacoustic Emissions

**Figure 1:**
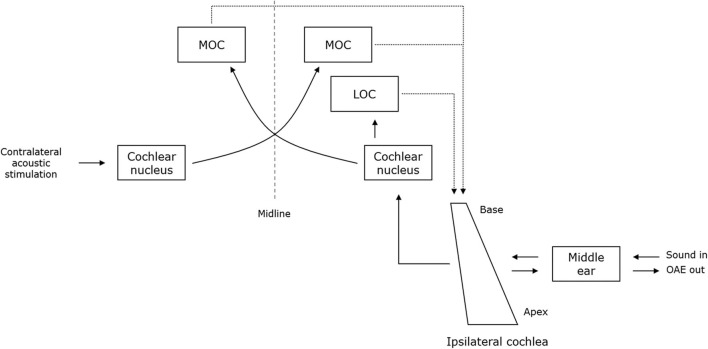
A schematic representation of the structures that are involved in the generation and suppression of OAEs. The dashed and full lines represent the afferent and efferent auditory pathways, respectively. Sound enters the ipsilateral cochlea through the middle ear and is analyzed along the length of the cochlea. Outer hair cells amplify the motion of the basilar membrane. Vibratory energy is transmitted backwards through the middle ear and produces the OAEs. The medial (MOC) and the lateral olivocochlear (LOC) system provide efferent innervation to the cochlea. The MOC system is stimulated by crossed afferents and projects to the ipsilateral and contralateral cochlea. MOC efferents have an inhibitory effect on OHC activity. Therefore, acoustic stimulation of the contralateral ear leads to a reduction of the response amplitudes of OAEs (i.e., efferent suppression). Based on Guinan (33).

Otoacoustic emissions (OAEs) provide a non-invasive and objective measurement of outer hair cell (OHC) functioning. OAEs are sounds of cochlear origin, which are recorded with a microphone probe fitted in the ear canal. The sounds originate from vibrations of the eardrum that are transmitted backwards from the cochlea through the middle ear. These vibrations are caused by the motion of OHCs as they respond to auditory stimulation (32) (Figure 1). There are two widely used OAE measurements: transient evoked OAEs (TEOAEs) and distortion product OAEs (DPOAEs). Robust TEOAE responses are evoked by click stimuli around 80 dB SPL. Although clicks are broadband signals, TEOAEs give a frequency specific indication of OHC functioning between 1 and 4 kHz. On the other hand, DPOAEs are evoked using pairs of pure tones at a lower stimulus level, usually 65/55 dB SPL. DPOAEs offer a wider frequency range than TEOAE measurements with less sensitivity to minor and subclinical alterations in adults. Similar to hearing thresholds, age-related changes in the OAE response can be expected (34). In routine audiological practice, it is the presence of a detectable OAE response to a particular stimulus that is important. Frequencies at which hearing thresholds exceed 35 dB HL typically show an absent OAE response. Hence, the presence of OAE decreases with increasing age, especially at higher frequencies. In patients with PD, the presence of TEOAE and DPOAE responses did not differ significantly from HCs (17, 20). Additionally, for research purposes, the response amplitudes of OAEs can be used for group comparisons. Regarding TEOAEs, two studies reported lower response amplitudes in medicated patients with PD compared to HCs (18, 23). In contrast, De Keyser et al. (17) found no significant differences regarding TEOAE response amplitudes between medicated patients with PD and age-matched HCs. Regarding DPOAEs, similar response amplitudes were found between medicated patients with PD compared to HCs (17, 23). Nevertheless, a modulatory effect of dopaminergic medication on the OAE response amplitudes has been found. OAE response amplitudes were higher when the same patients with PD were tested without their medication (17). This result corroborates with the study of Lopes et al. (19), who found higher DPOAE response amplitudes in patients with PD receiving low daily doses of dopaminergic medication compared to patients receiving higher doses.

Moreover, OAEs can be used to examine the integrity of the efferent auditory pathways by applying contralateral acoustic stimulation (CAS). In normally hearing individuals, OAE response amplitudes decrease during CAS, because acoustic stimulation activates the medial olivocochlear (MOC) efferent system, which in turn has an inhibitory effect on OHC motility. This inhibitory effect is termed efferent suppression (ES). Generally, if CAS fails to reduce the OAE response amplitudes by 1 dB or more, ES is described as abnormal (35). In PD, abnormal ES of TEOAEs has been reported by Di Mauro et al. (18). In this study, “increased TEOAE response amplitudes during CAS” were described in patients with PD. It is, however, unclear whether response amplitudes were increased compared to HCs or whether they were increased compared to response amplitudes of the same patients, but in the absence of CAS. In any case, this result indicates decreased to absent ES of TEOAEs in patients with PD, which may support the involvement of the efferent auditory pathway in the pathology of PD. Regarding ES of DPOAEs, both patients and HCs generally demonstrated adequate reduction during CAS and no significant difference could be demonstrated regarding the amount of ES between patients with PD and HCs (19). Nevertheless, a modulatory effect of dopaminergic medication dosage has been found on ES in patients with PD. ES of DPOAEs was higher in patients receiving higher daily doses of dopaminergic medication compared to those receiving lower doses, albeit only statistically significant at 2 and 3 kHz.

Regarding early stage PD, only Pisani et al. (23) evaluated OAEs in 11 *de-novo* patients with PD. In their study, OAEs were administered to investigate possible alterations of cochlear functioning after initiating dopaminergic treatment in previously untreated patients with PD. In the drug-naïve condition, lower TEOAE and DPOAE response amplitudes were found in patients with PD compared to HCs. Following the initiation of dopaminergic treatment for a period between 1 and 3 months, TEOAEs remained unchanged, while DPOAE response amplitudes significantly increased compared to the drug-naïve condition, resulting in an outcome more similar to HCs.

#### Auditory Reflexes

Stapedial reflex testing is usually administered as part of acoustic immittance measurements (tympanometry). When presented with a sufficiently high-intensity stimulus, the stapedial muscles contract bilaterally and stiffen the ossicular chain, thus decreasing middle-ear admittance (36). The stapedial reflex depends on the intact function of the entire reflex arc, including the middle and inner ear (sensory receptors), the cochlear nerve (afferent neurons), the lower brainstem (interneurons) and the facial nerve (efferent neurons). The stapedial reflex can be measured both ispilaterally and contralaterally. The specific site of lesion is determined by comparing the stapedial reflexes in response to ipsilateral vs. contralateral acoustic stimulation. The clinically most relevant outcome of stapedial reflex testing is the acoustic reflex threshold (ART) or the lowest intensity at which a minimal change of admittance is measurable. The ART generally ranges between 70 and 100 dB HL for pure tones. In the study of Murofushi et al. (37), patients with PD and HCs demonstrated an ART that was well within normal limits, although the ART of patients with PD was significantly lower compared to HCs. For research purposes, a series of latency measures can be extracted from the stapedial reflex. In patients with PD, the latency between stimulus onset and the time at which the stapedial reflex reaches 50% of its maximal amplitude was significantly prolonged compared to HCs (37). In addition, following cessation of the stimulus, patients with PD demonstrated a significantly longer latency for the amplitude to decrease to 50% of its maximal amplitude compared to HCs. These latency measures did not differ significantly between patients with PD taking dopaminergic medication and those who were not taking dopaminergic medication.

An auditory reflex that is also elicited by a high-intensity stimulus, is the auditory startle response (ASR). The ASR is a generalized motor response produced reflexively in response to a sudden, loud sound that results in a quick, usually observable movement. It is generally believed to be a brainstem reflex that originates in the pontine reticular formation, which is innervated by auditory afferents through the cochlear nucleus and connects with efferents that supply the target muscles, including the bulbar reticular tract, the reticulospinal tract, spinal interneurons and cranial and spinal motor neurons (38). The muscular activity of interest, such as the ocular, facial, neck, upper, and lower extremity muscles, can be objectively measured with electromyography (EMG). A variety of response parameters can be extracted from the EMG response, including the amplitude, latency, duration and habituation of the ASR. In the auditory field, the ASR is known as a cursory test for hearing sensitivity (39). In addition, an increased ASR has been associated with reduced sound tolerance (40), hyperacusis (41) and tinnitus (42). In PD, the ASR has primarily been studied as a potential marker for the differentiation from patients with progressive supranuclear palsy. The neural loss in this type of parkinsonism specifically involves the cholinergic neurons of the pontine reticular formation, including those involved in the ASR (43). In patients with PD, studies have generally found present ASRs demonstrating a normal amplitude, duration and habituation compared to HCs (43–46). Regarding the onset latency of the ASR, both prolonged (45, 46), as well as shortened latencies (44) have been reported in patients with PD, depending on the muscular activity of interest. Prolonged latency measures have been related to withdrawal of facilitatory input to brainstem centers from the basal ganglia (45, 46). No modulatory effect of dopaminergic medication has been found on the ASR in patients with PD (45, 46).

No study has specifically focused on auditory reflexes in early stage PD. While auditory reflexes may be able to differentiate between patients with PD and progressive supranuclear palsy in the clinical stages of PD, it appears unlikely that abnormalities regarding the latency of auditory reflexes will be able to reliably detect early stage PD. Furthermore, latency abnormalities have also been reported in other parkinsonian disorders, such as multiple system atrophy (44, 47) and dementia with Lewy bodies (44). These findings suggest that auditory reflex testing has limited potential to discriminate PD from atypical parkinsonian disorders. In addition, auditory reflexes are mediated by multiple neural circuits and abnormal or absent responses do not necessarily comprise a problem with auditory function.

### Central Auditory Tests

#### Psychoacoustic Experiments Assessing, Pitch, Loudness, Temporal Perception

Psychoacoustic experiments are concerned with the relationship between the physical characteristics of an auditory stimulus and their perceptual attributes (48). In PD, psychoacoustic research has been used to examine the perception of basic acoustic features (frequency, intensity, duration), i.e., pitch, loudness and duration. The methods used in these studies vary greatly according to stimulus type (verbal and non-verbal stimuli) and task demands (detection, discrimination, categorization and estimation). Regarding psychoacoustic experiments using pure-tone stimuli, patients with PD demonstrated increased discrimination thresholds for duration (49–52) and frequency [albeit not statistically significant (51, 53)] compared to HCs. Likewise, patients with PD had more difficulty at discriminating perceptually small acoustic differences compared to HCs (12, 54). On the other hand, when perceptually larger differences were used to assess auditory discrimination, no significant differences could be found between patients with PD and HCs (12, 53, 55). Auditory categorization and estimation tasks of intensity and duration have generally not found significant differences between patients with PD and HCs (53, 56–60). Regarding psychoacoustic experiments using speech stimuli, multiple studies have found abnormal perception of speech intensity (61–64). More specifically, patients with PD appear to overestimate the intensity of less intense speech and to underestimate the intensity of more intense speech.

Only a few studies have administered psychoacoustic experiments in patients with early stage PD. Breitenstein et al. (54) evaluated duration and frequency discrimination to assess the contribution of altered auditory perception to the disturbed perception of prosody. In this study, a subgroup of six recently diagnosed (on average 16.2 months) patients with PD were included who had not yet received dopaminergic treatment. No significant differences were found between early stage patients with PD and age-matched HCs for the discrimination of pure tones differing either with respect to frequency or duration. In the study of Lopes et al. (20), patients with PD were also divided into two subgroups regarding disease stage. As such, 34 patients with PD at H&Y stage I to II were included in the early stage PD group. The Duration Pattern Test demonstrated that early stage patients with PD had more difficulty to identify the order of a sequence of three pure tones (e.g., long—short—short) compared to HCs. This difference was, however, only significant in a subgroup of patients with PD aged 42–64 years, but not in older patients with PD. In the same study, the Gap-in-Noise test did not reveal significant differences between early stage patients with PD and HCs, as the duration detection threshold for silent intervals embedded in ongoing noise was similar in both groups. Likewise, patients in the more advanced PD group did not differ significantly from HCs. Lastly, Graber et al. (65) examined whether altered duration perception in PD affects their categorical perception of phonemes. They included nine early stage patients with PD, who were all at H&Y stage I to II and had a maximum disease duration of 6.0 years. The word-medial occlusion length and voice onset time were varied in order to create a continuum between two unambiguous endpoints, the minimal word pairs boden/boten and dick/tick, respectively. For both manipulations, early stage patients with PD made, on average, similar categorical decisions compared to age-matched HCs. Three patients, however, perceived “boden” throughout the whole continuum and needed a considerably longer occlusion interval to perceive the minimal pair cognate “boten.” This result could not be readily explained by differences regarding disease staging (as all patients were in an early disease stage), age, dopaminergic medication state, nor verbal intellectual abilities. Altogether, current research has found no evidence for alterations regarding the perception of basic acoustic features in early stage PD. In more advanced PD, psychoacoustic studies suggest that (ab)normal auditory perception is highly dependent on the paradigm that is used (16). More specific, tasks that probe the lower limits of the ability to detect and discriminate auditory changes have demonstrated abnormal results in PD. Further research might indicate whether more complex psychoacoustic experiments could be suitable to differentiate early stage patients with PD from HCs.

#### Dichotic Listening Tests

Dichotic listening tests are among the most widely used behavioral tests to assess central auditory processing. Dichotic listening refers to listening to different speech stimuli presented to each ear simultaneously or in an overlapping manner (66). A great variation of dichotic listening tests exists, varying with regards to the speech stimuli (i.e., consonant-vowels, digits, words or sentences), as well as the response condition. Two types of response conditions can be used that engage the integration or segregation of binaural auditory input, respectively (30). Namely one condition that requires divided attention (free recall condition) and one condition that requires selective attention (right/left ear recall condition). Multiple studies have addressed dichotic listening in a divided attention paradigm in patients with PD. Overall, these studies did not find significant group differences between patients with PD and HCs (20, 22, 67, 68). Only Richardson et al. (69) reported that a substantial part of patients with PD demonstrated abnormal dichotic listening compared to normative values.

In agreement with research in more advanced patients with PD, Lopes et al. (20) found no alterations regarding dichotic listening in a divided attention paradigm in early stage PD. In their study, a subgroup of 34 patients with PD at H&Y stage I to II did not differ significantly from HCs regarding the percentage of correctly repeated digits. On the other hand, Sharpe (70, 71) has evaluated dichotic listening in a more complex task to examine (auditory) attentional deficits in patients with early stage PD. The task consisted of dichotically presented word pairs containing the target word or a phonemic distractor paired with a phonetically unrelated word. Two studies used this task in either a divided attention paradigm (71) or a selective attention paradigm (70). In both studies, 14 patients with PD were included that were at H&Y stage I to II and had a mean disease duration of 4.2 years. In the divided attention paradigm, patients with PD discriminated significantly less target words compared to HCs. In the selective attention paradigm, patients with PD and HCs did not differ significantly regarding the discrimination of target words in the to-be attended ear, considering the percentage of ipsilateral responses to targets and distractors was similar in both groups. Nevertheless, the author suggested that “patients with PD were more prone to the interference of phonemic distractors,” as patients with PD made “more contralateral responses to false positive errors in the unattended ear.” In sum, most studies point to a preserved performance in conventional dichotic listening tests that probe divided auditory attention in all clinical stages of PD, including the earliest disease stage. Nevertheless, more complex dichotic listening tests may be able to differentiate early stage patients with PD from HCs.

#### Binaural Interaction Procedures

Binaural interaction tests depend upon intact binaural processing in the central auditory nervous system. Binaural processing enables sound localization and lateralization, and improves speech perception in adverse listening conditions. These tests require the combination of complementary input presented to both ears simultaneously, synthesizing intensity, temporal, and spectral differences of otherwise identical stimuli (66). Binaural interaction is presumed to occur in the brainstem (72). Hence, these tests are thought to be sensitive to brainstem lesions. A great variation of procedures can be used to evaluate binaural interaction. Some of these tests may be useful to demonstrate alterations regarding binaural processing in patients with PD compared to HCs, including auditory lateralization (73) and spatial listening tasks (22). In contrast, binaural masking level difference tasks, that assess the ability to detect a tonal stimulus in noise when the binaural signal-to-noise phase relationships are altered, show no significant differences in patients with PD compared to HCs (22).

No study has specifically focused on binaural auditory processing in early stage PD.

### Summary: Audiological Measurements in PD

A summary of study findings regarding audiological measurements in early stage patients with PD can be found in Table 1. Altogether, auditory research in PD has found alterations at all clinical disease stages using audiological measurements associated with both peripheral and central auditory processing. Unfortunately, to date, few studies have specifically examined auditory processing in early stage PD. Nevertheless, these studies suggest an involvement of the auditory system early in disease progression. At the peripheral level, three studies using subjective pure-tone audiometry provided evidence that hearing thresholds in the middle to high frequency range may be increased in early stage patients with PD. In one of these studies, hearing thresholds did not change following initiation with dopaminergic medication. Likewise, objective OAE measurements demonstrated altered auditory processing in early stage patients with PD, based on decreased response amplitudes compared to HCs. Moreover, OAE response amplitudes increased following initiation with dopaminergic medication. Although OAEs are generally associated with cochlear dysfunction, alterations may also indicate a dysfunction of the efferent control of the OHCs by the MOC system. In this regard, decreased ES of OAEs has been reported in patients with more advanced PD. However, further research is warranted into early alterations of the MOC system in PD, assessed using OAE and ES measurements. Especially since an involvement of the MOC system can be hypothesized during the early stages of PD pathology involving the lower brainstem (75). Behavioral measurements associated with central auditory processing in PD demonstrated that results are generally highly dependent on the paradigm that is being used. Therefore, experiments may have to be tailored toward specific hypotheses regarding early stage PD. For example, more complex tasks that stress the auditory system to a higher level, such as adaptive measurements of auditory discrimination and adapted tasks of dichotic listening, may be more suitable to discriminate patients with early stage PD from HCs.

**Table 1:** Summary of study findings regarding audiological measurements in early stage patients with Parkinson's disease.

**Audiological measurements**	**Description**	**Early stage PD studies**	**Study findings**	**Effect of dopaminergic medication**	**Strengths/limitations**
**Peripheral auditory tests**					
*i. Pure-tone audiometry*	Measurement of hearing sensitivity to pure-tone stimuli	(23*, 28, 74)	Higher hearing thresholds at 2, 4, 6, and 8 kHz	No effect was found	+ Gold standard in audiology, standardized clinical method – Low specificity (high prevalence of high-frequency hearing impairment in the general elderly population), subjectivity
*ii. Speech audiometry*	Measurement of speech recognition	No early stage PD studies	−	−	+ Information on functional auditory status – Time consuming, influence of cognitive factors, low sensitivity (most patients in the clinical PD stage demonstrate no abnormalities), subjectivity
*iii. Otoacoustic emissions*	Measurement of outer hair cell cochlear functioning	(23)*	Lower TEOAE and DPOAE response amplitudes in *de-novo* patients with PD	DPOAE response amplitudes increased	+ Objectivity, direct link to neurotransmission, time efficiency, standardized clinical method, ability to detect subclinical auditory alterations – Low specificity (reduction of OAE response amplitude in the general elderly population), absent response at hearing thresholds >35 dB HL
*iv. Auditory reflexes*	Measurement of stapedial reflex or auditory startle response	No early stage PD studies	−	−	+ Objectivity – Low specificity (atypical parkinsonian disorders demonstrate abnormal auditory reflexes as well)
**Central auditory tests**					
*i. Psychoacoustic tests*	Measurement of acoustic feature perception	(20, 54, 65)	No difference in gap-in-noise detection, frequency and duration discrimination, phoneme categorization, altered duration pattern recognition	−	– Time consuming, subjectivity, no standardized clinical method, low sensitivity (most patients with early stage PD demonstrate no abnormalities)
*ii. Dichotic listening tests*	Measurement of the integration or segregation of binaural auditory input	(20, 70, 71)	Divided attention paradigm: no difference in digit repetition, altered target word discrimination in a complex task Selective attention paradigm: no difference	−	+ Sensitized test (high level of difficulty) – Influence of cognitive factors, no standardized clinical method, subjectivity
*iii. Binaural hearing tests*	Measurement of the integration of intensity, temporal, or spectral differences of otherwise identical stimuli	No early stage PD studies	−	−	– No standardized clinical method, subjectivity

Study findings indicate the results of audiological measurements in early stage patients with Parkinson's disease (PD) compared to healthy control participants. Studies investigating the effect of dopaminergic medication on auditory processing in early stage PD are indicated with an asterisk (*). The main strengths and/or limitations whether or not to incorporate the audiological measurements into a prodromal auditory marker protocol for PD are given. PD, Parkinson's disease.

## Electrophysiological Measurements

### Short Latency Auditory Evoked Potentials

Short latency auditory evoked potentials (AEPs), better known as the auditory brainstem response (ABR), are routinely evaluated in audiological practice. In adults, the peak latency and interpeak latency (IPL) of the ABR are typically used in the diagnosis of retrocochlear pathologies, such as vestibular schwannoma and lesions of the brainstem. The ABR consists of seven distinct waves that occur within 10 ms and is recorded with a high-intensity transient acoustic stimulus, most commonly a click. The auditory nerve and auditory nuclei located in the brainstem are the major structures involved in the generation of the ABR. Each wave, labeled using Roman numerals, reflects the synchronous firing of different auditory cell populations. As such, waves I and II are derived from the distal and proximal regions of the auditory nerve as it enters the brainstem, while waves III to VII are generated from successively higher brainstem structures (76). The components following wave V show large intersubject and within-subject variability and are, therefore, less useful for research purposes. Studies on age-related changes of the ABR have reported only slight changes regarding wave latencies and IPLs. Generally, prolongations appear to be limited to the early waves I to III, suggesting that age primarily affects peripheral auditory nerve transmission (77, 78). As increasing evidence suggests that PD initially affects the brainstem and follows a predominant upwards course (75), considerable attention has been devoted to measures of brainstem functioning in PD, such as the ABR. On the one hand, various studies have reported normal latency measures in patients with PD compared HCs (25, 27, 79, 80). On the other hand, multiple other studies have reported prolonged latencies for the centrally generated waves III to V in patients with PD compared to age-matched HCs (21, 24, 28, 81–84). In addition, prolonged IPLs have also frequently been found in patients with PD compared to HCs for waves III-V, and I-V. The differences between study results cannot be readily explained by methodological differences or patient variables. At present, although there is some evidence that suggests abnormal ABR results, experimental support for altered auditory brainstem processing in patients with PD is inconsistent.

Regarding early stage PD, two studies have investigated the ABR in early stage patients with PD. Karayanidis et al. (79) administered ABR testing in 16 patients with PD (to ensure that the main outcome of their study, which was centered around the long latency event-related potentials (ERPs), was not confounded by differences in auditory brainstem processing). All patients had a relatively recent diagnosis (on average 3.0 years) and “most of them were at H&Y stages I or II”. Compared to age-matched HCs, patients with PD exhibited a non-significant prolongation of the IPL between wave I and III. No significant group differences were found for the IPL between wave I and V and wave V latency. In contrast, Yylmaz et al. (28) found significantly prolonged IPLs between wave I and V and wave V latencies in 20 patients with PD at H&Y stage II compared to HCs. However, in their study, patients with PD were on average 6.1 years older compared to the HC group. The result of Yylmaz et al. (28) may indicate a similar pattern of centrally located ABR abnormalities found in more advanced patients with PD. Nonetheless, further research is warranted.

### Middle Latency Auditory Evoked Potentials

Following the ABR, middle latency AEPs are generated between 10 and 50 ms after the onset of a transient auditory stimulus, such as a tone burst or a click. Middle latency AEPs are not routinely administered in audiological practice, as they are highly sensitive to the participant's attention and state of arousal, as well as to several recording parameters, especially stimulus presentation rate (85–87). However, when these variables are adequately controlled for, middle latency AEPs can be useful to assess the functional integrity of the auditory pathways and to localize lesions at the thalamocortical and primary auditory cortex levels (87). Traditionally, four positive and three negative peaks are included in the middle latency AEPs, namely V, N0, P0, Na, Pa, Nb, and P50. In patients with PD, P50 is the most frequently studied middle latency AEP. When using a sufficiently low stimulus presentation rate, no abnormalities regarding P50 amplitude or latency appear evident in patients with PD compared to HCs (88–91). On the other hand, higher presentation rates may lead to P50 being absent or having a prolonged latency in a substantial part of patients with PD (92). In addition to stimulus trains, the P50 can be evoked by pairs of stimuli. The paired-stimulus paradigm is useful for investigating auditory gating. Auditory gating represents the central auditory nervous system's ability to suppress irrelevant auditory stimuli (93). When two identical stimuli are presented in a paired-stimulus paradigm, P50 to the second stimulus is inhibited relatively to the first stimulus in HCs. Patients with PD exhibited significantly less inhibition of P50 to the second stimulus compared to age-matched HCs, suggesting diminished auditory gating in PD (90, 91). All patients were in rather advanced disease stages (H&Y ranging between III and V). Interestingly, when patients were divided according to disease stage, both studies found that patients at H&Y stage III did not differ significantly from HCs regarding P50 inhibition, and that abnormalities were limited to patients with PD at H&Y stages IV and V.

No study has specifically focused on the middle latency AEPs in early stage PD. It may be interesting to investigate middle latency AEPs in early stage patients with PD in response to high stimulus presentation rates, as abnormalities were found in a large sample of patients with PD (n = 46) that varied greatly regarding H&Y disease stage (range I to IV) and disease duration (range 1–24 years) (92). Regarding auditory gating, based on the studies of Teo et al. (90, 91), it appears unlikely that P50 inhibition would be able to discriminate early stage patients with PD from HCs, given the finding that only patients in the most advanced disease stages differed significantly from HCs in terms of P50 inhibition.

### Long Latency Auditory Evoked Potentials

#### P1-N1-P2 Complex

The auditory P1-N1-P2 complex represents an exogenous stimulus-related response associated with sound detection (94, 95). The ERP-waveform consists of three deflections, namely P1, N1, and P2 that reach their maximal amplitude at around 50, 100, and 160 ms respectively. The first component, P1, has been considered to overlap -to some extent- with the P50 studied as a middle latency AEP. The auditory P1-N1-P2 complex can be evoked in both passive or active listening conditions based on different stimulus paradigms, such as stimulus trains, paired-stimulus paradigms or oddball paradigms. In audiological practice, the P1-N1-P2 complex has been most commonly investigated as an objective counterpart of behavioral audiometry to estimate a participant's hearing thresholds. On average, the P1-N1-P2 complex can be found at 10 dB above the behavioral hearing threshold (96). From a broader point of view, the outcome of the P1-N1-P2 waveform highly depends on the acoustic characteristics of the stimulus, such as frequency, intensity, duration, and location (94). Based on the majority of studies that used an auditory oddball paradigm, no altered auditory P1-N1-P2 complex in patients with PD compared to HCs could be found (16). Significant differences in latency or amplitude values of the different subcomponents have been reported between patients with PD and HCs [e.g., (97–101)], however, no conclusive pattern of auditory alterations emerged. It is unclear which participant, clinical or ERP related variables could explain the heterogeneous study results.

Regarding early stage PD, the same inconclusive pattern was found (79, 99, 100, 102–107). Therefore, no clear alterations in stimulus-related sound detection in the early stage of PD can be assumed based on the current results from auditory oddball paradigms. However, in the study of Beucke et al. (108), altered intensity dependence of AEPs (IDAEP) was demonstrated in early stage PD. A significantly increased IDAEP of the N1/P2 amplitude, indicating low serotonergic activity, was found in unmedicated patients with PD compared to HCs. This difference was no longer evident after 12 weeks of dopaminergic treatment in patients with PD (108). In addition, based on a paired-stimulus paradigm, Lukhanina et al. (109) and Lukhanina et al. (110) demonstrated significantly reduced post-excitatory inhibition of the auditory N1/P2 complex following the second stimulus in patients with PD evaluated without dopaminergic medication state compared to HCs. Subgroup analyses based on disease stage, revealed that diminished post-excitatory auditory cortical inhibition may be evident at the early stage of the disease (110). Furthermore, auditory inhibition of the second stimulus seems to improve after dopaminergic intake in patients with PD (110).

#### Mismatch Negativity

The auditory mismatch negativity (MMN) is an ERP associated with the automatic pre-attentive detection of a deviant auditory stimulus in a sensory memory trace (85, 111, 112). As such, the MMN may be considered as a signature of auditory discrimination abilities (94, 113). The component is classically obtained with an auditory oddball paradigm in which a deviant stimulus infrequently occurs in a sequence of standard stimuli. The deviant stimulus may comprise a change in frequency, duration, intensity, location, inter-stimulus interval, or the omission of the stimulus compared to the standard stimulus (85, 113, 114). The component is very useful in clinical conditions that require no cooperation of the patient since the MMN can be elicited in the absence of the participant's attention (113). Accordingly, the participant can be asked to perform a visual task, such as watching a silent-video or reading a book, whilst ignoring the auditory stimuli, or to focus on stimulus characteristics other than that of the deviant stimulus during an attentive oddball paradigm (113, 115, 116). The MMN response is detectable with a deviant-minus-standard wave and peaks between 100 and 250 ms at the frontocentral and central scalp electrodes. Based on the review by Seer et al. (117) no evidence was found for altered MMN latency and amplitude values in non-demented patients with PD compared to HCs. In addition, no effect of dopaminergic medication on the auditory MMN could be demonstrated (115, 116).

In most of the studies, the auditory MMN was evaluated in (subgroups of) patients with early PD (79, 99, 115, 116, 118). Thus, it can be assumed that no deficiencies in automatic auditory discrimination are present in the early stage of PD as investigated with a MMN paradigm, irrespective of the dopaminergic medication status (115). Furthermore, no significant staging effect on the MMN was found in non-demented patients with PD in a later study of the same research group (116). Decreased auditory MMN amplitudes may be evident when PD is associated with PD dementia and hence, neurodegeneration is in an advanced stage (116, 118).

#### Processing Negativity or Nd

In selective attention tasks, attended and unattended auditory stimuli differ, for example, in location, frequency (i.e., pitch) or both on which basis the participant may select the task-relevant stimuli (96, 119, 120). More specifically, the participant attends one channel in which a deviant stimulus must be detected in a sequence of standard stimuli whilst ignoring the other channel (oddball paradigm in a dichotic listening condition) (119). Generally, the ERPs related to the standard stimuli in the attended channel are compared to those of the unattended channel. The negative shift of the ERPs to the attended stimuli relative to the unattended stimuli has been related to selective auditory attention and has been identified as the Nd or processing negativity (PN) (119, 121–123). Based on decreased amplitude values of the Nd in patients with PD, alterations in selective auditory attention in PD have been suggested (79, 106, 124). However, it remains unclear which aspects of selective auditory attention might be altered in patients with PD, as different results have been reported regarding the early central subcomponent (Nd1) and the late frontal subcomponent (Nd2) (16). The Nd1 may reflect the matching process of incoming auditory stimuli with the internal template, whereas Nd2 is thought to represent the updating of the internal template (125). Nonetheless, further research is needed since selective auditory attention has not yet been investigated sufficiently based on electrophysiological dichotic listening paradigms. Yet, investigating this aspect of auditory processing might be highly relevant even more because alterations of the Nd have been demonstrated in studies in which early stage PD patients were included (79, 106, 124). Vieregge et al. (124) evaluated the effect of dopaminergic medication on the Nd in patients with PD with an average disease duration of 5.0 years and H&Y stages ranging between I and III. Compared to HCs, a decreased Nd amplitude was reported in patients with PD after a 12-hour withdrawal from dopaminergic medication. Following dopaminergic medication, the Nd remained unchanged in patients with PD.

#### N200

The auditory N200 or N2 is a negative wave between 200 and 350 ms post stimulus onset that is endogenous in nature (96, 126). Different components of the N2 wave have been described based on the design of the ERP paradigm and its modality, namely N2a, anterior N2 (N2b) and posterior N2 (N2c) (85, 126). An auditory N2a can be elicited by an inattentive auditory mismatch effect in which case the component is commonly known as the MMN as described above (85, 126). Regarding the N2b component, latency and amplitude values have been defined based on the deviant target stimuli in attentive auditory oddball paradigms. In this regard, various studies have reported an increased N2 latency in non-demented patients with PD compared to HCs [e.g., (97, 101, 127)] although the result was not always statistically significant and non-differences have also been demonstrated [e.g., (105, 128)]. Most studies found no differences in N2 amplitude between patients with PD and HCs [e.g., (101, 105, 127)]. Yet, a decreased N2 amplitude has been reported in the studies of Lagopoulos et al. (129), Lagopoulos et al. (98), and Pekkonen et al. (99) using an auditory oddball paradigm. Regarding the N2c component, PD related results are beyond the scope of the current review since this component has been specifically considered for the visual modality.

Regarding early stage PD, Broussolle et al. (130) evaluated N2 latency in 8 *de-novo* patients with PD with an average disease duration of 1.3 years. Six of these patients had not yet received dopaminergic medication. N2 latency did not significantly differ between patients with PD compared to age-matched HCs. Likewise, the majority of studies reported no abnormalities regarding N2 latency in patients with early stage PD, neither without dopaminergic medication (102), nor with dopaminergic medication (79, 99, 105, 107). Only Philipova et al. (100) found a prolonged N2 latency in patients with PD compared to HCs, but only when participants were instructed to provide a motor response to the presented stimuli and not when they were required to count the target stimuli. Overall, however, no clear N2 alterations can be assumed early in disease progression.

#### P300

The auditory P300 or P3 is a high-level endogenous cognitive ERP generated by an attentive response to an infrequent deviant stimulus (131). Usually, the distinction is made between a parietally maximal P3b component and a frontally maximal P3a component. The P3b component is elicited after the presentation of a task-relevant deviant stimulus and may be considered a signature of auditory categorization based on top-down voluntary goal-driven attention and working memory processing (132, 133). In addition, the P3a is elicited by a non-target task-related or novel distractor stimulus and can be regarded as a neurophysiological marker of bottom-up involuntary stimulus-driven attention and the orienting response (134–137). The auditory P3 component has been classically obtained with a two (standard, deviant) or three-stimulus (standard, target deviant, non-target deviant/novel distractor) auditory oddball paradigm in which the participants are instructed to count the task-relevant deviant stimuli or to respond by a button-press. The P3 can be described by the average of the deviant stimuli or a deviant-minus-standard wave and starts from 250 ms at the parietal and frontal scalp electrodes for the P3b and P3a respectively. Regarding P3b, the review of Seer et al. (117) concluded that a prolonged P3b latency may be evident in demented patients with PD. The authors found no conclusive pattern of P3b differences in non-demented patients with PD, although P3b latency was significantly increased in 38% of the related studies. In addition, it was stated that P3b amplitude was generally found to be unaltered in demented and non-demented patients with PD (117). However, it should be noted that regarding the P3b specifically, both auditory and a smaller amount of visual oddball paradigms were considered. Regarding P3a, latency and amplitude findings were rather heterogeneous (117), although a decreased P3a amplitude seems to be related to disease duration (116, 117).

Regarding early stage PD, significant differences in latency or amplitude values of the P3b component have been reported in patients with PD compared to HCs (100, 103–105, 107, 138). Overall, however, no clear pattern of P3b alterations emerged in the early stage of cognitively non-impaired patients with PD (79, 100, 102–105, 107, 130, 138–141). In addition, no effect of dopaminergic medication could be shown in the study of Georgiev et al. (140). In contrast, evidence has been demonstrated for alterations of the P3a component in early stage patients with PD compared to HCs (15, 104, 115, 116, 138, 140, 142). In the study of Cavanagh et al. (142), a trend for an increased P3a amplitude was found in early stage patients with PD. The authors suggested that—when filter settings are considered—the altered P3a amplitude is in line with the study of Solis-Vivanco et al. (116) in which a decreased P3a amplitude could be demonstrated in patients with PD compared to HCs. Moreover, a diminished habituation to novel stimuli over time was found in the PD group (142). Likewise, alterations of the P3a amplitude were evident in the study of Pauletti et al. (138). In their study, an increased P3a latency and decreased P3a amplitude were shown in PD patients with central fatigue (138). Although no significant differences in the P3a component were found between patients with PD and HCs in the study of Solis-Vivanco et al. (15), the authors suggested that impaired novelty detection may be evident in the early stage of PD based on a reduced phase alignment for deviant stimuli using time-frequency based analyses (15). Finally, amplitude alterations of the P3a component seem most evident when patients with PD are evaluated with dopaminergic medication (115, 140, 142).

### Summary: Electrophysiological Measurements in PD

A summary of study findings regarding electrophysiological measurements in early stage patients with PD can be found in Table 2. Based on various AEPs, neurophysiological alterations in auditory processing have been demonstrated in the clinical stage of PD. Considering early stage PD, a careful selection of electrophysiological paradigms may be suitable to discriminate patients with early stage PD from HCs. Study results particularly suggest a pattern of centrally located ABR abnormalities (wave III–V) in PD. However, as ABR studies in early stage PD are limited and rather inconsistent, further research is warranted. Especially because of the potential involvement of the lower brainstem during the early stages of PD pathology (75). Furthermore, altered long latency AEPs in early stage PD may be evident when ERP paradigms are tailored to evaluate specific and/or more complex auditory processes. Regarding paired-stimulus and intensity dependence paradigms, an increased N1/P2 amplitude in early stage patients with PD has been found. These differences disappeared following initiation with dopaminergic medication in patients with PD. Regarding selective attention and three-stimulus oddball paradigms, studies have shown a decreased Nd amplitude and a decreased or increased P3a amplitude. Regardless of the direction of the P3a amplitude alteration, shifts of the P3a component were most evident when patients with PD were evaluated with dopaminergic medication.

**Table 2:** Summary of study findings regarding electrophysiological measurements in early stage patients with Parkinson's disease.

**Electrophysiological measurements**	**Description**	**Early stage PD studies**	**Study findings**	**Effect of dopaminergic medication**	**Strengths/limitations**
Short latency AEPs	Measurement of auditory brainstem responses	(28, 79)	Non-significant prolonged IPL I–III, no difference or prolonged IPL I–V and wave V latency	–	+ Objectivity, standardized clinical method, established neural generators, high specificity – Inconsistent evidence
Middle latency AEPs	Measurement of subcortical and primary auditory cortex responses	No early stage PD studies	–	–	+ Objectivity – No standardized clinical method, low sensitivity (most patients in the clinical PD stage demonstrate no abnormalities)
**Long latency AEPs**					
*i. P1-N1-P2*	Measurement of auditory signal detection	(79, 99, 100, 102–107, 108*, 109, 110*)	Increased IDAEP of the N1/P2 amplitude Decreased inhibition of the N1/P2 amplitude using a paired-stimulus paradigm No clear alterations using an auditory oddball paradigm	IDAEP differed no longer with HCs Inhibition of the second stimulus increased	+ Objectivity, information on distinct auditory (sub-)processes, direct link to neurotransmission, correlated with behavioral outcome measures – Influence of subject related variables, such as alertness and arousal, low sensitivity for specific ERP components (e.g., MMN, N2 and P3b)
*ii. MMN*	Measurement of automatic pre-attentive auditory discrimination	(79, 99, 115*, 116*, 118)	No clear alterations	No effect was found	
*iii. Nd/PN*	Measurement of auditory selective attention	(79, 106)	Decreased Nd amplitude	–	
*iv. N2*	Measurement of voluntary auditory discrimination and categorization	(79, 99, 100, 102, 104, 105, 130)	No clear alterations	–	
*v. P3*	Measurement of attentive auditory categorization related to (1) bottom-up involuntary stimulus-driven attention and the orienting response (P3a) or to (2) top-down voluntary goal-driven attention and working memory processing (P3b)	(15, 79, 100, 102–105, 107, 115, 116, 130, 138, 139, 140*, 141*, 142*)	Evidence for P3a alterations No clear P3b alterations	P3a: Increased P3a amplitude P3b: No effect was found	

Study findings indicate the results of electrophysiological measurements in early stage patients with Parkinson's disease (PD) compared to healthy control participants. Studies investigating the effect of dopaminergic medication on auditory processing in early stage PD are indicated with an asterisk (*). The main strengths and/or limitations to whether or not incorporate the electrophysiological measurements into a prodromal auditory marker protocol for PD are given. PD, Parkinson's disease; AEPs, auditory evoked potentials, IPL, interpeak latency; IDAEP, intensity dependence of auditory evoked potential; MMN, mismatch negativity; PN, processing negativity.

## Discussion

Prodromal markers are defined as indicators of an ongoing neurodegenerative process in the central or peripheral nervous system prior to the typical symptoms allowing a clinical diagnosis (143). A prodromal marker can refer to any disease indicator (1), whether it be of clinical, neuro-imaging, biochemical or genetic origin. Of critical importance in the utility of prodromal markers is their sensitivity (the certainty with which a marker can identify prodromal PD) and specificity (the ability of a marker to identify disease-free individuals). In addition, the practicalities and difficulties involved in assessing a prodromal marker must be considered (10). More specifically, a prodromal marker should be non-invasive, easy and relatively inexpensive to administer, sufficiently available, and reliable (9, 10).

Ideally, studies that aim to define prodromal markers should be prospective and prodromal markers should be identified before the patients develop PD (10). Unfortunately, to date, no prospective studies have assessed the potential of auditory markers in the prodromal stage of PD. Nevertheless, indirect evidence for the relevance of auditory markers in prodromal PD comes from studies in which auditory assessment was carried out in the early clinical disease stage. At present, multiple studies have investigated clinically diagnosed patients with early stage PD (H&Y ≤ II). H&Y stage I and II represent early pathology in the olfactory bulb and the lower brainstem (75), possibly causing prodromal symptoms (e.g., olfactory dysfunction, constipation and rapid eye-movement sleep behavior disorder) (144). As such, the earliest pathological disease stages are an important step in delineating which auditory measurements may be useful to detect prodromal PD. However, whether audiological or auditory electrophysiological measurements have the potential to detect prodromal PD depends on the necessary characteristics of a biomarker. Overall, auditory alterations in PD can be evaluated with relatively low-cost and non-invasive audiological and electrophysiological measurements that are already routinely available in clinical practice.

Regarding audiological measurements associated with peripheral auditory processing, pure-tone audiometry revealed significantly increased middle to high frequency hearing thresholds in early stage patients with PD compared to HCs. However, increased pure-tone hearing thresholds are highly prevalent in the elderly population, which seriously affects the specificity of hearing thresholds as a marker of prodromal PD. In addition, pure-tone audiometry is a behavioral test that relies on a subjective response and requires repeated responses to provide a reliable outcome. Hence, the potential of pure-tone audiometry as a marker of prodromal PD is rather limited. An objective measurement that is generally associated with peripheral auditory processing involves OAEs. OAEs are relatively easy to acquire, fast to administer and require limited cooperation of the participant. Research has found preliminary evidence for altered OAE response amplitudes in *de-novo* patients with PD. Moreover, OAEs have been reported to be sensitive to dopaminergic medication, which might make them relevant for monitoring the pharmacological response. However, older patients, as well as patients with a history of excessive noise exposure will potentially produce rather weak OAEs with no measurable responses in the high frequency range. Hence, OAE measurements in PD comprise both strengths and shortcomings regarding biomarker perspectives. Therefore, further research into the sensitivity and specificity of OAE alterations in prodromal PD is warranted.

The involvement of the peripheral auditory system in PD has generally been regarded as an age-dependent sensorineural dysfunction (i.e., presbycusis) (23, 27). Structural changes underlying presbycusis include loss of cochlear hair cells, which in turn, affects afferent transmission of auditory information to higher auditory structures (145). Cochlear hair cells are subject to feedback control from the olivocochlear efferent system, which has been proposed to play an important role in preventing noise-induced and age-related hearing impairment. The auditory efferent system originates in the brainstem and involves two major pathways. The LOC efferents provide innervation of the inner hair cells (IHCs), whereas the MOC efferents primarily project to the OHCs. Interestingly, dopamine is released from the LOC efferents and exerts a neuroprotective circuitry for the cochlea by preventing excitotoxic damage during glutamate overstimulation (146). In addition, alpha-synuclein has been located in the cholinergic MOC system (147). By directly controlling OHC motility and subsequently modulating basilar membrane motion, the MOC system is proposed to exert a second efferent pathway preventing hearing impairment. In sum, peripheral auditory alterations in PD most likely represent the combined effects of physiological aging processes and the neuropathological changes intrinsic to PD, which may leave patients with PD at a higher risk for developing noise-induced, as well as age-related, hearing impairment. However, the effect of dopaminergic medication on OAEs cannot be readily explained by dopaminergic expression at the level of IHCs, as OAEs mainly represent OHC function. Despite the apparent lack of dopaminergic terminals in the OHC region, Pisani et al. (23) suggested that dopamine may exert a modulatory effect on OHCs via LOC synapses on the MOC efferents. Alternately, De Keyser et al. (17) argued that abnormal OAEs might result from a dopamine deficiency at the level of the brainstem.

Audiological measurements associated with central auditory processing are inherently subjective in nature (66) and, therefore, have limited potential as a reliable marker for prodromal PD. In addition, in contrast to peripheral auditory tests, these measurements are no standardized audiological methods, using a variety of stimuli and procedures, and differing greatly regarding task demands. Generally, central auditory processing has been linked to behavioral phenomena such as sound localization, auditory discrimination and auditory performance with competing auditory signals (148). Interestingly, many of these central auditory processes can be objectively and possibly more sensitively assessed using electrophysiological measurements.

Auditory electrophysiological measurements are characterized by a high temporal resolution and provide a continuous measure of auditory processing, whereas behavioral measures of central auditory processing reflect the combined effect of many neural processes (85). Therefore, ERPs have the ability to demonstrate how auditory processing unfolds over time and to uncover which distinct neural processes may be altered in prodromal PD. For example, in contrast to behavioral measures of selective auditory attention, electrophysiological registration during dichotic listening tasks may reveal which specific stages in the process of selective auditory attention are altered in prodromal PD. Moreover, electrophysiological measurements meet the necessary practicalities of a biomarker. At present, electrophysiological studies of auditory processing in PD have primarily focused on the processing of non-verbal auditory stimuli using two-stimulus oddball paradigms. Based on early stage PD studies, auditory ERPs derived from this type of paradigm (e.g., MMN, N2, and P3b) seem unlikely to detect prodromal PD. Nevertheless, paradigms that assess specific and/or more complex auditory processes have demonstrated differences between early stage patients with PD and HCs. ABR measurements are well-known in clinical neurological practice and may be relevant to further investigate the potential early involvement of the lower brainstem in PD pathology (75). Nonetheless, bilateral central ABR prolongations have been reported in a wide variety of neurological conditions (e.g., demyelinating diseases, Alzheimer's disease, schizophrenia) (74, 149, 150) which may affect the specificity of ABR measurements as a marker of prodromal PD. Regarding intensity dependence paradigms, an increased IDAEP of the N1/P2 component has been found in early stage patients with PD compared to HCs suggesting lower serotonergic activity and related depression in PD (108, 151). Interestingly, symptoms of depression may occur before the clinical onset of PD (152, 153) and have already been prospectively established as a prodromal marker of PD (3). As such, IDAEPs are highly relevant as an objective indicator for this prodromal marker in PD. Finally, ERP paradigms that assess the modulation of incoming auditory stimuli have shown alterations in early stage PD compared to HCs ranging from automatic pre-attentive to attentive auditory processing. More specific, pre-attentive auditory gating abnormalities in early stage PD have been suggested given a disinhibited N1/P2 component in paired-stimulus paradigms. At a higher level, Nd and P3a component abnormalities have been shown using dichotic oddball or attentive three-stimulus paradigms in early stage PD. Therefore, electrophysiological measurements focusing on these aspects of auditory processing may have a potential as a marker for prodromal PD. However, further research is needed to detect and validate the AEPs that show abnormalities in the prodromal stage of PD.

The current study results may suggest an involvement of the central auditory system in early stage PD when involuntary inhibitory or attentive selective auditory processing is required. Along the auditory pathway, nuclei of the brainstem auditory system have been found to exert an inhibitory function in auditory processing (154). Since PD pathology especially involves brainstem structures during the early disease stages (75), inhibitory dysfunction of auditory processing in early stage PD can be hypothesized. Regarding higher-order auditory processing, the involvement of a dysfunctional fronto-striatal circuitry in attentive auditory processing in PD has been considered. To date, the pathophysiological mechanisms underlying central auditory processing deficits in PD are poorly understood and thus, are subject for further research. Nonetheless, dopamine deficiencies may be related to both auditory dysfunctions at the brainstem and cortico-subcortical level. Dopamine has been suggested a neurotransmitter involved in sensory processing and therefore, may be hypothesized to have a modulatory role in auditory processing. In the current review, study results may imply a positive effect of dopaminergic medication on inhibitory function of repeated auditory stimuli. In contrast, increased resource allocation for the unattended channel or novel (distractor) stimuli processing was suggested in early stage PD. Moreover, alterations of the P3a component were most evident when patients with PD were evaluated with dopaminergic medication. Since the cortico-subcortical circuits are not equally affected by PD pathology, dopaminergic medication may differentially effect higher-order cognitive auditory processing (155). Taken together, dopaminergic medication could modulate both peripheral and central auditory processing in patients with PD. However, few studies have directly investigated the effect of dopaminergic medication on auditory processing in early stage PD.

Overall, the current review provided evidence for auditory alterations in the early stages of PD. At present, however, the relevance of auditory markers in prodromal PD is unclear. In order to truly gain insight into the value of prodromal auditory markers, prospective studies in large populations or in selected high-risk groups are needed. The assessment of auditory processing is already routinely carried out in the general population and compromises standardized and reliable measurements, making them highly suitable for prospective biomarker studies.

## Conclusion

At present, research has demonstrated altered auditory processing in early stage PD using audiological and electrophysiological measurements. Future perspectives for auditory markers in the prodromal stage of PD can be found in the use of objective audiological and specific electrophysiological measurements. However, further research is warranted to assess the sensitivity and specificity of these auditory measurements as well as their relationship to other markers of prodromal PD. Since few studies have directly investigated the effect of dopaminergic medication on auditory processing in early stage PD, it is currently unclear whether patients with PD would benefit from early pharmacological intervention regarding auditory processing.

## Author Contributions

ED and KD wrote the manuscript under the supervision of MD. PS, DT, AB, DB, and MD provided critical feedback and helped shape the manuscript. All authors approved the final version for submission.

## Conflict of Interest

The authors declare that the research was conducted in the absence of any commercial or financial relationships that could be construed as a potential conflict of interest.
